# Common inputs to motor units are modulated by force profile and muscle length

**DOI:** 10.1113/EP094031

**Published:** 2026-08-29

**Authors:** Abdulkerim Darendeli, Oscar Soto, Eugene Tunik, Mathew Yarossi

**Affiliations:** ^1^ Department of Physical Therapy, Movement, and Rehabilitation Science Northeastern University Boston Massachusetts USA; ^2^ Department of Neurology Tufts Medical Center Boston Massachusetts USA; ^3^ Department of Electrical and Computer Engineering Northeastern University Boston Massachusetts USA

**Keywords:** dimensionality reduction, electromyography, motor neuron, muscle force, neural modules, rate of force development, size principle, synergy

## Abstract

Groups of motor units receive common synaptic inputs from specialized networks of premotor neurons to produce movement. Here, we test whether common inputs received by motor units in the first dorsal interosseous are fixed or task‐dependent during submaximal isometric contractions. Discharge times of motor units were identified and tracked across a set of conditions. Factor analysis identified a single motor unit mode that captured most of the covariation in the discharge rates of motor units, presumably reflecting common synaptic inputs. This was supported by the high correlation values between motor unit pairs from the same participant. Next, displacement analysis on the discharge rates of motor units was computed to capture the magnitude of departures from a monotonic control structure expected from fixed common inputs. For the same force profile, activity of motor units was limited to a set of discharge rate–time trajectories across trials, suggesting a constrained pattern of premotor inputs to motor units. Importantly, changes in force profile and muscle length modulated synaptic inputs to motor units, as observed in the divergent rate–time trajectories of motor units and high displacement values. These findings highlight that common input is a key feature in the neural control of motor units and is subject to substantial modulation. The composition of inputs to motor units depends on the force profile and muscle length, and the system relies on reusing a common input structure when performing the same task.

## Abstract (highlights)

- **What is the central question of this study?**
  There is extensive literature supporting that sets of motor units receive common synaptic inputs during static isometric contractions, but are common inputs fixed across a range of task conditions even within a single action like finger abduction?
- **What is the main finding and its importance?**
  For the same task condition, activity of motor units was limited to a set of discharge rate–time trajectories across trials, suggesting a constrained pattern of premotor inputs to motor units. Changes in force profile and muscle length, however, modulated synaptic inputs to motor units. We attribute this to changes in the distribution of common inputs, motor neuron membrane properties and neuromodulation of persistent inward currents.

## 1 INTRODUCTION

Movement is produced by the coordinated activity of neuronal populations including supraspinal, spinal and sensory afferent systems, and the final common pathway of this motor infrastructure is the motor unit, comprising a spinal motor neuron and the muscle fibres it innervates. The existence of multiple descending tracts (i.e., corticospinal, reticulospinal and other drives) with distinct roles (DeLong & Strick, 1974; Glover & Baker, 2022, Lemon, 2008), heterogeneous subsets of interneurons (Ampatzis et al., 2014; Chapman et al., 2025) and feedback from sensory afferents with their axons outnumbering motor axons by at least 9:1 (Gesslbauer et al., 2017) suggests a high neural degrees of freedom. On the other hand, local interneurons serve as integrators by routing most of the supraspinal and sensory inputs to motor units (Lesser et al., 2024). Despite the complexity of the motor system, studies suggest that the activity of motor units is ultimately constrained by common inputs, regardless of premotor neuronal source (Clamann & Henneman, 1976; Levine et al., 2023; Somjen et al., 1965).

How motor units are recruited to produce the large repertoire of voluntary behaviours observed in humans remains a fundamental question. Observation of highly correlated discharge across motor units is thought to result from shared synaptic currents, and has been used to support theory suggesting common synaptic inputs to motor units. Furthermore, common synaptic input provides the mechanistic basis for the size principle of motor unit recruitment, which states that input resistance alone determines recruitment order only when all motor units receive the same synaptic drive (Heckman & Enoka, 2012; Henneman et al., 1965b; Lesser et al., 2024). Indeed, multiple investigations have demonstrated that motor units exhibit strongly correlated discharge times, orderly recruitment and constrained rate–time trajectories amongst motor unit pairs during isometric muscle contractions in humans (Bräcklein et al., 2022; Cabral et al., 2025; Del Vecchio et al., 2023; Levine et al., 2023; Rossato et al., 2024). Moreover, during sustained contractions, a few low‐dimensional modes account for most variance in motor unit discharge rates, supporting the common input theory (Del Vecchio et al., 2023; Hug et al., 2021, 2023; Latash, 2020).

Existing human studies provide evidence for common inputs, but not whether these inputs are fixed (issued with minimal change in premotor neuron activity) or systematically modulated across task conditions. A recent study in macaques demonstrated that task conditions modulate motor unit pair discharge rate–time trajectories (Marshall et al., 2022), presumably reflecting changes in the distribution of inputs received by motor units. Interestingly, in fish there is a preferential synaptic input current to larger motor neurons (Kishore et al., 2014) during fast swimming indicating that recruitment order may be influenced by speed‐dependent modulation of synaptic inputs in addition to the intrinsic motor neuron properties (Gabriel et al., 2011). They noted that as swimming speed increases, excitatory drive to larger motor neurons preferentially increases, while inhibitory drive to all motor neurons uniformly increases regardless of their size. Similarly, in the fruit fly, while synaptic inputs to leg motor units scale with motor neuron size, input weights to wing‐steering motor units scale unrelated to motor neuron size (Lesser et al., 2024). Additionally, the composition of motor unit modes may be reorganized following a brief muscle stretch intervention, likely mediated by changes in afferent feedback (Weinman et al., 2024). These findings suggest the existence of significant modulation of common inputs to motor units.

To investigate whether the distribution of common inputs to motor units are modulated across a range of task conditions in humans, we analysed motor unit behaviour during isometric index finger abduction tasks involving different rates of force development, at long and short muscle lengths. We leveraged classical approaches to quantify the strength of common inputs by analysing the covariation in motor unit discharge rates, and a novel metric to identify violations of monotonicity expected from fixed common inputs (Marshall et al., 2022). We hypothesized that a single motor unit mode will account for most of the covariation in the discharge rates of motor units when performing an isolated task such as static contraction. Accordingly, the rate–time trajectories of motor unit pairs will be nearly fixed, indicating constrained common inputs, across trials within each isolated force profile to simplify the control of motor units. By contrast, we also hypothesized that motor unit discharge rate–time trajectories will diverge as a function of task condition and muscle length, indicating deviation from a fixed common input structure. Importantly, understanding the transmission of information from premotor neurons to motor units has implications for how the sensorimotor system executes behaviour, adapts to neurological injury, and will inform the development of diagnostic measures and neuroprosthetics.

## 2 METHODS

### 2.1 Ethical approval

The study was conducted in accordance with the *Declaration of Helsinki*. All protocols were approved by Northeastern University Institutional Review Board (no. 15‐10‐22). Ten healthy men (25.8 ± 6 years old) were recruited for the study. All subjects self‐reported having no diagnosed medical conditions and provided written informed consent after study protocols were verbally explained to them. All subjects self‐reported being right‐hand dominant.

### 2.2 Experimental design

**FIGURE 1:**
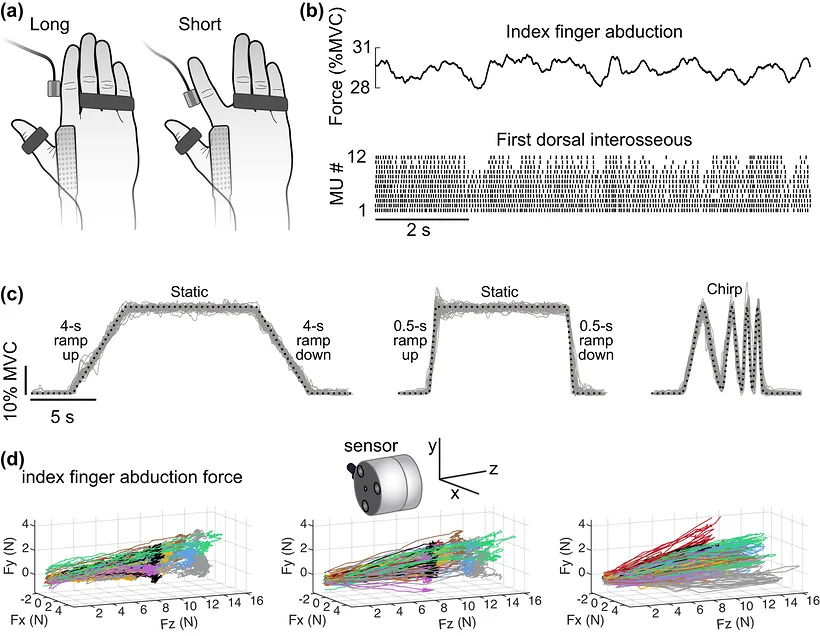
A view of experimental approach. (a) Subject hand postures during isometric finger abduction tasks with the first dorsal interosseous at a long (left) or short (right) length. The load cell made contact with the first interphalangeal joint of the index finger, either in neutral position (muscle length long) or with the index finger abducted at 30° (short muscle length). (b) Example applied force and decomposed single motor unit discharge times identified in the first dorsal interosseous. (c) Single trials (grey) across multiple subjects and instructed target (dotted black) forces at 30% MVC. (d) Three‐dimensional representation of force vectors. The three panels in (d) correspond, from left to right, to the three panels in (c). Each colour corresponds to a different subject. Directional cosine of the resultant force with respect to *F_z_* indicated that >95% of the applied force was in the abduction direction.

Subjects sat in a chair with their right arm loosely restrained to armrest (51 × 17 cm flat surface). The third, fourth and fifth digits were immobilized together using a Velcro strap applied along proximal phalanges and fixed to the armrest. The right hand was positioned palm‐down, with the thumb fixed at a 45° angle of palmar abduction. A load cell (six‐axis force transducer; Nano 17, DAQ F/T; ATI Industrial Automation, Apex, NC, USA) was firmly positioned to make contact with the first interphalangeal joint of the index finger to record finger abduction force during isometric muscle contractions (Figure 1a). Subjects were positioned ∼1 m away from a screen at eye level. Subjects received visual feedback from the screen displaying time‐varying force applied to the load cell. As a recent report suggests that the type of feedback (i.e., visual or auditory) may impact the processing of afferent inputs (Henry et al., 2025), the primary source of feedback and entire subject posture were kept consistent throughout the recording session.

Custom MATLAB (R2023b, MathWorks, Natick, MA, USA) scripts were designed to set relative target force levels, present instructions and operate hardware. Subjects were asked to match a red dot with a black target line on a screen located in front of them. The vertical position of the red dot was proportional to the force registered by the force sensor. The instructed target force moved leftward on the screen at a constant speed. To normalize force amplitude of subsequent tasks, maximal voluntary contraction (MVC) force during index finger abduction was recorded with the index finger in neutral position (long muscle length). Each MVC trial lasted ∼6 s and involved a 3 s increase from rest to maximum that was held for 3 s. There was 90 s of rest between trials. Each subject performed three trials, and the difference in peak force between the two highest MVCs was always <5%. The greatest MVC force was kept as the maximal value. Strong verbal encouragement was provided during each MVC trial. Subjects were familiarized with the experiments by performing a series of submaximal isometric contractions with the index finger abductors.

High density electromyography (HD‐EMG) signals were recorded from the first dorsal interosseous, with a grid of 64 electrodes (model GR04MMI305, 4 mm interelectrode distance, OT Bioelettronica, Turin, Italy). An adhesive foam layer was placed over the electrodes and filled with conductive paste (Ten20, Weaver and Co., Aurora, CO, USA) to ensure skin–electrode contact. The skin over the muscle was cleaned with sandpaper and water before placing grid of electrodes. Two wet (water) wrist straps with clip connectors were placed around the forearm as ground and reference electrodes. HD‐EMG signals were recorded in monopolar derivation with a sampling frequency of 2048 Hz and band pass filtered (10–500 Hz) using a multichannel acquisition system with a 16‐bit resolution (Quattrocento, OT Bioelettronica). Force exerted by the index finger was measured with a load cell (a six‐axis force transducer) and sampled at 1000 Hz (USB‐6218, National Instruments, Austin, TX, USA). One of the six‐axis force outputs was split and sent to the HD‐EMG acquisition system for offline synchronization.

### 2.3 Force profiles and muscle lengths

To investigate whether the distribution of common synaptic inputs received by motor units is invariant or modulated by task parameters, we examined discharge patterns across a set of conditions that systematically varied force profile and muscle length. Each force profile (e.g., static or chirp) and muscle length (long or short) was defined as a ‘condition’. These conditions were expected to influence motor unit activation through changes in premotor neuronal drive (DeLong & Strick, 1974; Marshall et al., 2022). Each subject completed six task blocks, three at long muscle length and three at short muscle length, with a 90 s rest between each block. A task block comprised two trapezoidal contractions, one with slow ramps and the other with fast ramps, and one chirp contraction with 30 s rest between each contraction. Force profile conditions included: a 4 s ramp up to target force, a 10 s static, a 4 s ramp down to resting, a 0.5 s ramp up to target force, another 10 s static, a 0.5 s ramp down to resting, and a so‐called chirp that sweeps from 0.33 to 1.25 Hz across four segments (0.33, 0.67, 1.25 and 1.25 Hz). Maximum target force was 30% MVC (Figure 1c).

Muscle length of the first dorsal interosseous was systematically varied by fixing the index finger in either a neutral position (long) or 30° of abduction (short). Muscle length was varied to modulate proprioceptive feedback to motor units (Cabral et al., 2024), and study the effect on the invariance of synaptic input.

### 2.4 Motor unit decomposition

The monopolar EMG signals were band‐pass filtered (10–500 Hz; fourth‐order, bidirectional Butterworth) and each of the 64 channels was visually inspected. Channels with artifacts or low signal‐to‐noise ratio were removed. The HD‐EMG signals were decomposed into motor unit spike trains using a convolutive blind source separation technique (Negro et al., 2016) implemented in open‐source MATLAB software (MUedit, v1.2) based on fast independent component analysis (Avrillon et al., 2024). The automated process first adds 1000 delayed versions of each channel and whitens the extended channels. Then, a fixed‐point algorithm iteratively optimizes the separation vector (initialized with random weights) using a cost function ‘skew’ (*g*(*x*) = *x* ^2^) to maximize the sparsity of estimated motor unit pulses. Once the fixed‐point algorithm terminates, *k*‐means and peak detection functions separate the high peaks (spikes) from low peaks (noise) in the estimated motor unit pulse trains.

The HD‐EMG signals recorded during the three submaximal contractions (30% MVC) within each task block were first decomposed individually. Subsequently, the signal sets were concatenated, and the separation vectors (also known as motor unit filters) from the decomposition were applied to the entire signal to track motor units. Duplicate motor units were automatically removed. When the discharge times of two motor units were ≥30% in common, it was considered a duplicate, and the motor unit with the higher coefficient of variation for interspike intervals was removed (Holobar et al., 2010).

The decomposition results were manually inspected by iteratively re‐estimating the spike trains with the separation vectors and correcting for missed spikes (Avrillon et al., 2024; Del Vecchio et al., 2020). The normalized distance between the spikes and the noise (silhouette value) was used as a measure of decomposition accuracy, with the values ranging from 0 to 1, where 1 indicates perfect accuracy. A silhouette value of ≥0.9 was used as a criterion for considering a motor unit for further analysis (Negro et al., 2016). However, initial decomposition results that met this criterion were not immediately accepted. All motor unit pulse trains were visually inspected and checked for errors. After the manual inspection and editing of motor unit discharge times, we checked for and removed duplicate motor units once again.

### 2.5 Motor unit tracking

**FIGURE 2:**
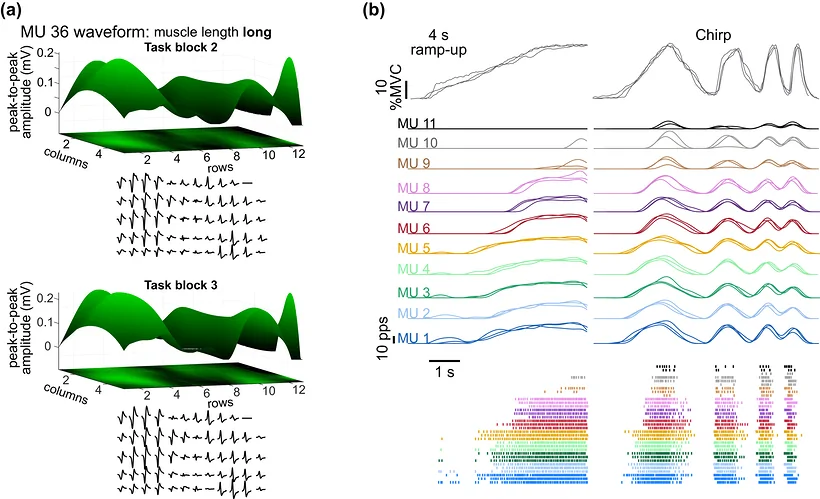
Example motor unit waveforms and responses across force profiles and trials from an individual representative subject. (a) Spike‐trigger averaged action potential waveforms (single‐differential), and their three‐dimensional representation of peak‐to‐peak amplitudes for an example motor unit tracked across task blocks. Motor units were tracked across tasks using the separation vectors (from the decomposition) and two‐dimensional correlation of spike‐trigger averaged motor unit action potential waveforms (cross‐correlation value = 0.96). (b) Motor unit discharge times (raster plots, one row per trial) and smoothed discharge rates (three trials superimposed) during three 4 s ramp up (left column) and chirp (right column) trials. Each colour corresponds to a different motor unit. Vertical scale bars are 10% MVC or 10 pulses per second (pps), and horizontal scale bar is 1 s.

We tracked single motor units across force profiles and muscle lengths to observe changes in the metrics used in our study. To achieve this, we applied motor unit separation vectors from the decomposition to signal sets within and across the task blocks. Secondly, we estimated spike‐trigger averaged motor unit action potential waveforms that were obtained from single differential derivation of the signal and computed the two‐dimensional correlation of waveforms. After visual inspection by an investigator, motor unit waveforms with a correlation coefficient greater than 0.85 were accepted as being from the same motor unit (Figure 2a). There were no instances where a motor unit waveform was matched with multiple others above a correlation coefficient greater than 0.85. Each motor unit was assigned a unique identifier. Both methods allow reliable tracking of motor units (Goodlich et al., 2023).

### 2.6 Data analysis

Each binary motor unit spike train was converted into a smoothed discharge rate by convolving it with a 1 s Hann window (Weinman et al., 2024). This smoothing time‐scale was chosen to capture overall trends in motor unit discharge rates while minimizing rapid fluctuations, providing conservative estimates of monotonic relationships between motor unit pairs across conditions. Force signals were low‐pass filtered (fourth‐order, bidirectional Butterworth with 20 Hz cut‐off) and temporally aligned with the motor unit data.

Motor unit recruitment thresholds and discharge rates during 4 s ramp up contractions were computed to assess the level of agreement for orderly recruitment within long and short muscle lengths. These variables were not used to infer changes in the distribution of synaptic inputs received by motor units. Recruitment threshold was identified as the index finger abduction force (%MVC) at which the first action potential was discharged within a 0.5 s window where the coefficient of variation for interspike intervals was less than 50%. This criterion ensures that transient discharges are not assigned as the first discharge. Discharge rate at recruitment was identified as the smoothed discharge rate corresponding to the first action potential (Pascoe et al., 2011). Instantaneous discharge rate of motor units was computed by taking the inverse of interspike intervals.

### 2.7 Motor unit discharge rate displacement

We used a *displacement* metric previously developed by Marshall et al. (2022) to quantify the flexibility of motor unit activation using experimental data. It captures the magnitude of departures from the hypothesis that the motor neurons receive a monotonic shared command from the premotor neurons (i.e., descending, sensory, intersegmental and interneuron). Of note, classical studies describe common input as synaptic currents shared across multiple motor units (Del Vecchio et al., 2023; Farina & Negro, 2015; Hug et al., 2021; Lesser et al., 2024). In this study, we investigated whether the distribution of these inputs remains constant (fixed common inputs) or changes (variable common inputs) across conditions or trials.

In a scenario where a common input is sent to a population of motor units, when the discharge rate of one motor unit changes, the discharge rate of all others in the same pool should change in the same direction (increase or decrease). Assuming that the population state (discharge rate) at time *t* is denoted as *r_t_* = [*r*_1,_ *_t_ r*_2,_ *_t …_ r_n_*_,_ *_t_*] (where *r_n_*_,_ *_t_* is the discharge rate of the *n‐*th motor unit), when the discharge rates of all motor units increase or saturate, this suggests an increase in the common input from *t* to *tʹ*. Conversely, if the discharge rates of all motor units decrease, it suggests a decrease in the common input. If coordinated changes in motor unit discharge rates are driven exclusively by scaling of a fixed common input, it implies a monotonic relationship between all motor unit pairs. This is a generous assumption biased in favour of the common input hypothesis in that it does not quantify the correlation networks of motor unit discharge rates to infer about shared inputs. Rather it only aims to capture deviations from a single, monotonic input signal.

**FIGURE 3:**
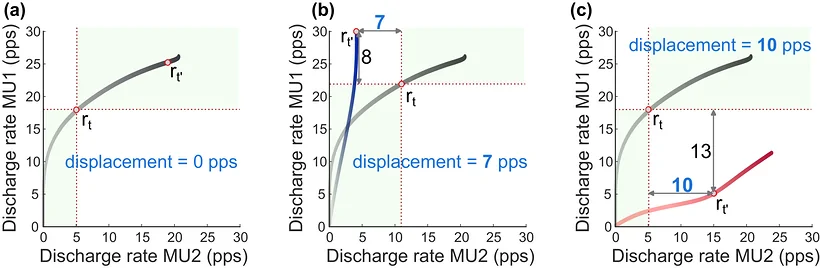
Quantification of motor unit displacement. Magnitude of displacement for discharge rates of two motor units at two times (*r_t_* and *r_tʹ_*) shown in three scenarios. The assumption is that if a pair of motor units receives common inputs, their discharge rate should change monotonically. The displacement metric quantifies the magnitude of departures from a monotonic path. (a) As the discharge rates of motor units 1 and 2 change monotonically (increase or decrease together) from *t* to *tʹ*, displacement must be 0. Any monotonic trajectory passing through *r_t_* is constrained to the green‐shaded zone. (b) The closest a monotonic manifold passing through *r_t_* can get to *r_tʹ_* is 7 pulses per second (pps) away. (c) *r_t_* cannot reach *r_tʹ_* by only increasing or decreasing the discharge rates of both motor units. The closest a monotonic path can get is 10 pps away.

The displacement metric first computes the largest non‐negative change in discharge rates between *t* and *t*ʹ, across all time points for each possible combinations of motor units. This was done by quantifying Δ*r* ^+^(*t*, *tʹ*) = max(0, *r*_1_ *_,t_* − *r*_1_ *_,tʹ_*, *r*_2,_ *_t_* − *r*_2,_ *_tʹ_*) and Δ*r* ^+^(*tʹ*, *t*) = max(0, *r*_1,_ *_tʹ_ − r*_1,_ *_t_*, *r*_2,_ *_tʹ_ − r*_2,_ *_t_*). Next, the departure from a monotonic trajectory was computed as *D*(*t*, *tʹ*) = min (Δ*r* ^+^(*t*, *tʹ*), Δ*r* ^+^(*tʹ*, *t*)). If the discharge rate changes monotonically, either Δ*r* ^+^(*t*, *tʹ*) or Δ*r* ^+^(*tʹ*, *t*) will be zero. Consider the example in Figure 3a: where discharge rates of two motor units at two time points are *r_t_* = (5, 18) and *r_tʹ_* = (19, 25). Here, Δ*r* ^+^(*t*, *tʹ*) = 0, and Δ*r* ^+^(*tʹ*, *t*) = 7, and therefore *D*(*t*, *tʹ*) = 0.

Finally, we introduced ±25 ms time lags to compare time points not just at the same instant, but also within the lagged window around that time to account for slight delays. We took the minimum value across the time lagged displacement results. This ensures that modest departures from monotonicity cannot be attributed to latencies in detection of motor unit discharges. The displacement was computed for three sets of data: (1) within the 4 s ramp up (representing within force profile) for each muscle length, (2) across all force profiles for each muscle length, and (3) across all force profiles and muscle lengths. Data were concatenated across the force profiles and the two muscle lengths. All possible pairs of simultaneously recorded motor units were included in the analysis. For each participant, we then took the median displacement value for each of these three categories for visualization. We also report the cumulative density function of displacement values for each pair.

The displacement metric was designed to quantify the smallest possible violation of monotonicity. Consider the example in Figure 3b, where discharge rates of two motor units at two time points are *r_t_* = (11, 22) and *r_tʹ_* = (4, 30). A one‐directional manifold passing through *r_t_* cannot reach *r_tʹ_* by monotonically increasing or decreasing the discharge rate. Two potential explanations were proposed by Marshall et al. (2022): First, common input increased (hence the increase in MU1's rate) but MU2 received a private inhibitory input (hence the decrease in MU2's rate). Second, the common input considerably decreased, but the MU1 received a private excitatory input. This would require a larger departure from monotonicity compared to the first scenario (8 vs. 7 pulses per second, pps). In both cases, there is a departure from a monotonic path, and the displacement metric quantifies the magnitude of this departure by selecting the smaller one (7 pps). An alternative scenario could involve changes in the distribution of common inputs from various sources, such as differential weighting of distinct premotor neurons.

We made the following decisions to quantify the displacement metric conservatively. (1) Each motor unit spike train was converted into a smoothed discharge rate by convolving it with a 1 s Hann window. This relatively long time scale smoothing was chosen to capture the overall trend in motor unit activity while minimizing the influence of rapid fluctuations. This ensures that modest departures from monotonicity cannot be attributed to rapid rate modulations. (2) The largest non‐negative change in discharge rates was computed bidirectionally, and the minimum value at time *t* was taken. (3) If this value was greater than zero, ±25 ms time lags were introduced at around both *t* and *tʹ*, and displacement was computed across these windows. Then, displacement at time *t* was considered as the minimum value found across these time‐lagged computations.

### 2.8 Factor analysis

The invariance of common synaptic inputs can be inferred by measuring covariation in the smoothed discharge rates of concurrently active motor units. Applying dimensionality reduction techniques, such as factor analysis, to these discharge patterns has revealed low‐dimensional structures known as motor unit modes (Del Vecchio et al., 2023). Before asking whether the distribution of common inputs to motor units is modulated across task conditions, we first tested whether our dataset confirmed previous findings that control of motor units is constrained and can be represented by a single mode when performing static contractions.

We performed factor analysis on the discharge rates of first dorsal interosseous motor units identified during a 10 s static contraction at 30% MVC for each subject individually using the *factoran* function in MATLAB. The 10 s static force trial with the greatest number of identified motor units among the six task blocks performed by each subject was used for the analysis. Each binary motor unit spike train was converted into a smoothed discharge rate by convolving it with a 1 s Hann window (Weinman et al., 2024). Factor analysis models the associations between these discharge rates into a lower number of factors. It assumes that a set of factors explain the common variance in the observed variables. If we performed the factor analysis on a concatenated dataset of all the force profiles (e.g., 4 s ramp, chirp), the assumption of cov(*x*) = ΛΛ^T^+Ψ, where ΛΛ^T^ is the common variance matrix and Ψ = cov(*e*) is the diagonal matrix of specific variances would be violated, as we assumed that the input structure to motor units would vary across force profiles (Marshall et al., 2022). Therefore, we performed factor analysis within a single force profile. We used at least four concurrently and continuously discharging motor units to perform a factor analysis. The mode with the most explanatory power for the common variance was referred to as motor unit mode 1. We then computed pairwise correlations between smoothed discharge rates of individual motor units for each subject to estimate the relative proportion of common synaptic input (Farina et al., 2016; Levine et al., 2023).

### 2.9 Statistical analysis

R (version 4.4.1; R Foundation for Statistical Computing, Vienna, Austria) was used for all statistical analyses. We compared linear mixed effects models with various fixed effects and covariates using ANOVA tests. We favoured models where added predictors significantly improved model fit, while maintaining a similar level of complexity. The following tools for assumption testing were used: (1) quantile–quantile and lag plots for residuals, (2) R performance package for visual inspection of normality of random effects, homogeneity of variance, heteroscedasticity and model‐predicted data fits, and (3) influential observation (outlier) check based on Cook's distance method with a threshold of 0.5 (Cook, 1977). Significance was set at 0.05 for all statistical analyses.

Motor unit data were not averaged within a factor for any analysis. We fit a linear mixed effects model (lme4 package) using Satterthwaite's method to investigate changes in displacement metric, with a fixed effect of condition (three levels: within force profile, across force profiles, across force profiles and muscle lengths) and a random intercept for each subject. We chose to quantify displacement during a 4 s ramp to represent the *within force profile* condition. No significant outliers were detected in the displacement data. The model‐predicted data captured the main distributional features of the observed data, and normality of random effects was confirmed. However, the heteroscedasticity assumption was violated. Therefore, we log‐transformed the displacement data before fitting the model (Displacement ∼ 1 + Condition + (1 | Subject)). *P*‐values were adjusted with Tukey's method for paired comparisons (emmeans package). Displacement is a recently developed metric and its association with the established methods to estimate the strength of common synaptic inputs, such as pairwise correlations between smoothed discharge rates of individual motor units, is unknown. We modelled this association by applying repeated‐measures correlation (*R*_rm_; rmcorr package) between motor unit displacement (across all force profiles) and correlation of smoothed discharge rates during 10 s static contractions (Bakdash & Marusich, 2017). *R*_rm_ determines within‐subject association for paired measures while accounting for non‐independence of observations.

To investigate changes in motor unit recruitment threshold, discharge rate at recruitment and maximum instantaneous discharge rate during a 4 s ramp up, as a function of muscle length, we fit separate linear mixed effects models, with a fixed effect of muscle length (long, short), and random intercepts for subjects and for motor units nested within subjects (e.g., Rec. Threshold ∼ 1 + Muscle length + (1 | Subject:MU)). No significant outliers were detected. The model‐predicted data captured the main distributional features of the observed data. Normality of random effects and heteroscedasticity assumptions were met. Magnitude of differences in motor unit recruitment threshold, discharge rate at recruitment and maximum instantaneous discharge rate between the muscle lengths was quantified using Cohen's *d* effect size. The magnitude thresholds were: |*d*| < 0.2 negligible, |*d*| < 0.5 small, |*d*| < 0.8 medium, and |*d*| ≥ 0.8 large.

Repeatability for motor unit recruitment thresholds across three trials per muscle length was assessed using intraclass correlation coefficient (ICC(3,1); two‐way, mixed model, absolute agreement) and its 95% confidence intervals (CI). The magnitude thresholds were: ICC < 0.5 poor agreement, 0.5 ≤ ICC < 0.75 moderate agreement, 0.75 ≤ ICC < 0.90 good agreement, and ICC ≥ 0.9 excellent agreement. Note that this analysis does not quantify recruitment threshold‐based ranking of motor units across trials.

## 3 RESULTS

### 3.1 Force production task and motor unit decomposition

Each of the 10 subjects completed six task blocks (three at long muscle length and three at short muscle length) with each task block comprising two trapezoidal contractions (one with slow ramps and the other with fast ramps) and one chirp contraction (a total of 18 contractions per subject). Index finger abduction forces closely matched the instructed targets (Figure 1c). Directional cosine of the resultant force with respect to *F_z_* verified that most of the force was applied in the abduction direction (Figure 1d; average cosine(*F_z_*) = 0.979). Motor units were successfully identified in 54 out of 60 task blocks. A total of 349 motor units were identified and tracked across all subjects and force profiles. On average 5.8 ± 3.9 motor units per task block were identified and tracked in each task block across all force profiles for each subject. A total of 83 unique motor units were tracked across all force profiles and the two muscle lengths (see Figure 7h).

### 3.2 Motor unit activity within and across force profiles

**FIGURE 4:**
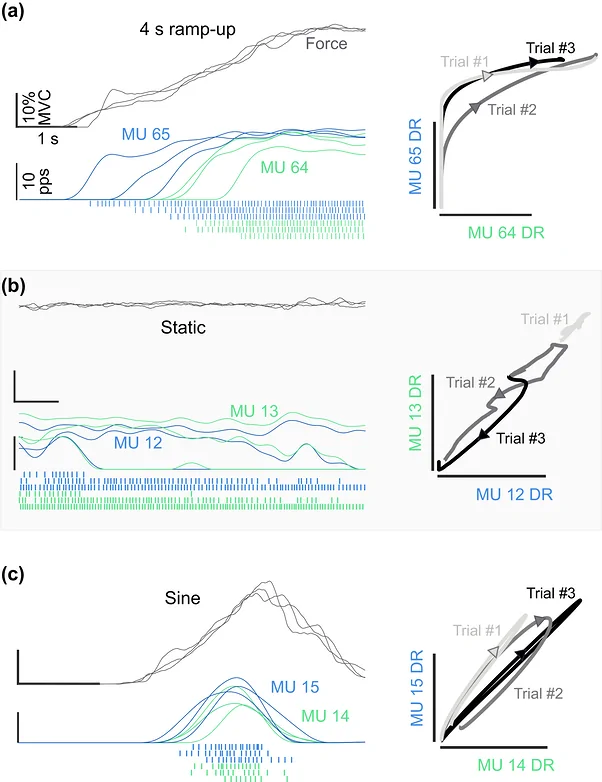
Common inputs constrain the activity of motor units to a set of discharge rate and time within each force profile. Left column, discharge times of a pair of motor units (bottom), their smoothed discharge rate (middle), and index finger abduction force (top) for 4 s ramp‐up (a), static (b), and first period of chirp (c) force profiles across three trials. Spike rasters (one row per trial) and smoothed discharge rates (three trials superimposed) are coloured by motor unit within each panel. Vertical scale bars are 10% MVC or 10 pulses per second (pps), and horizontal scale bars are 1 s. Right column, discharge rates of the same pairs of motor units, represented in state space, for the three force profiles. Motor unit activity is restricted to a space across the three trials (shaded light to dark grey) within each force profile. Scale bars are 10 pps.

State‐space trajectories of motor unit pairs were visually inspected across different force profiles, including static, 4 s ramp, 0.5 s ramp and chirp periods. Importantly, these trajectories followed consistent, largely monotonic patterns within each force profile, with no substantial violations across trials (see examples in Figure 4). To quantify departures from a monotonic trajectory, we chose to compute displacement during 4 s ramp up (representing *within force profile* condition). State‐space trajectories can be non‐linear, but larger drive should increase or saturate the discharge rates of motor units. This was consistent with our observations. For example, in Figure 4a, motor unit 65 starts discharging during a 4 s ramp up and as its rate saturates, another motor unit (64) becomes active. In the second trial, a larger force yank at the onset of the ramp induces some change in the state‐space trajectory, causing a small displacement. However, the discharge rate–time trajectories remain largely monotonic and constrained.

For the 4 s ramp condition, 94.2% of all displacement values were less than 2 pps (*n* = 2752 pairs; see Figure 6c,d), similar to those observed in non‐human primate motor units (Marshall et al., 2022). These observations suggest that common premotor projections constrain motor unit activity to a limited set of discharge rate–time trajectories during the execution of a certain force profile. This is consistent with the studies that tested the common input hypothesis using dimensionality reduction or coherence analysis of identified motor units during single force profile (static) tasks (Cabral et al., 2024; Del Vecchio et al., 2023; Hug et al., 2021). Inspection of representative examples can be useful to propose explanations. Consider the motor unit responses during static force profile, in Figure 4b, for example. Although the level of force was similar across the three trials, motor units (12 and 13) discharged tonically during the first trial but were active only briefly at the beginning of the third trial. Importantly, their state‐space trajectory remained monotonic across the three trials, consistent with the common input hypothesis.

**FIGURE 5:**
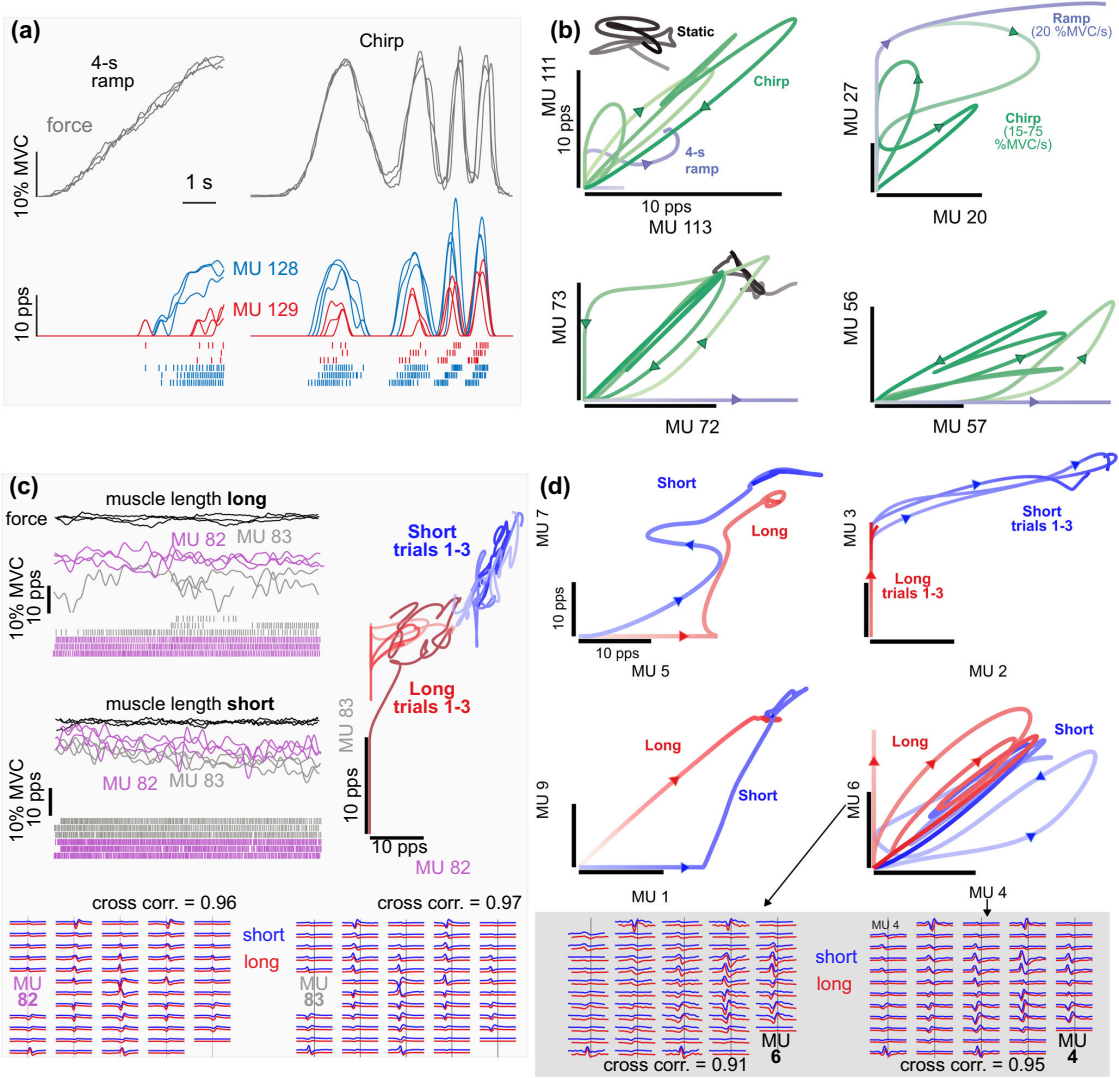
Changes in force profile and muscle length modulate the distribution of synaptic inputs to motor units. (a) Example discharge times of a pair of motor units (bottom), their smoothed discharge rate (middle), and index finger abduction force (top) for 4 s ramp up and chirp force profiles. Spike rasters (one row per trial) and smoothed discharge rates (three trials superimposed) are coloured by motor unit within each panel. (b) Discharge rates for pairs of motor units, represented in state space, across different force profiles (colour‐coded). Line colours get darker with time. Scale bars are 10 pps. (c) As in (a) but across muscle lengths. State space plot for the same motor units is shown on the right. (d) As in (b) but within force profile and across muscle lengths (colour‐coded). For clarity, each state‐space plot in (c) and (d) displays a single force profile. However, all force profiles and muscle lengths were included in the computation of displacement. Spike‐trigger averaged action potential waveforms (single‐differential), and two‐dimensional cross‐correlation values for the example motor units tracked across the muscle lengths are demonstrated below panels (c) and (d).

There was a significant main effect of condition (i.e., within force profile, across force profiles, and across muscle lengths) on displacement (*P* < 0.0001). Displacement values were significantly higher when all force profiles were included (mean ± SD: 6.9 ± 2.2 pps) compared with the within‐force profile condition (mean ± SD: 0.7 ± 0.9 pps; *P* < 0.0001, Tukey‐adjusted). Motor unit responses (Figure 2b; Figure 5a) differed between slow ramp and chirp segments, despite matched force amplitudes. This was previously noted (Büdingen & Freund, 1976; Del Vecchio, 2023). In Figure 5b, we show discharge rates for pairs of motor units, represented in state space, for 4 s ramp up, static and chirp force profiles. State space trajectories diverged from monotonic manifold with the addition of force profiles, leading to substantial violations of monotonicity. Rather than ruling out the common input hypothesis, this suggests that common inputs to motor units are task‐dependent and adapt to meet changing task demands.

### 3.3 Motor unit activity across muscle lengths

**FIGURE 6:**
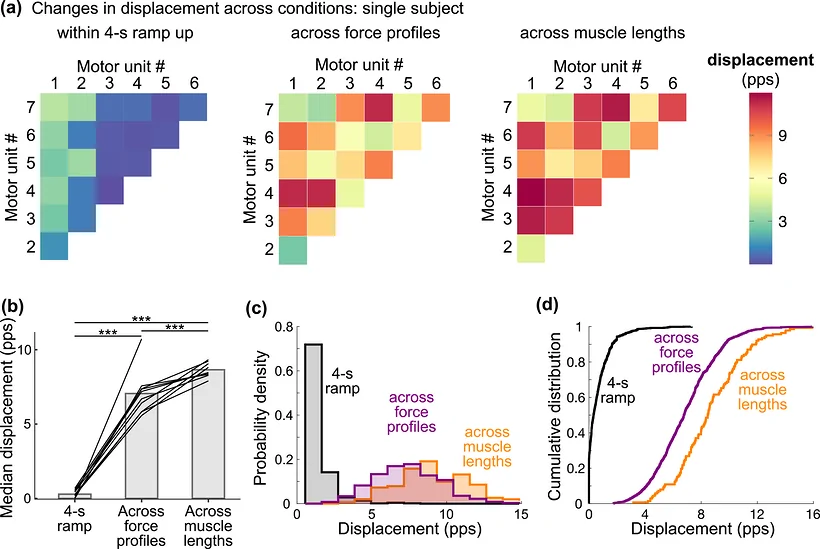
Displacement was computed (1) within the 4 s ramp up (representing within force profile), (2) across all force profiles within a muscle length, and (3) across all force profiles and muscle lengths. (a) Changes in displacement values across the three condition categories in a representative subject. (b) Median displacement for each subject (one line per subject). Median displacement values significantly increased with the inclusion of all force profiles and further increased when muscle lengths were also considered (main effect of condition and all Tukey corrected pairwise comparisons *P* < 0.0001). (c, d) Density (c) and cumulative distribution (d) of displacement for 4 s ramp (black), across force profiles (purple) and across muscle lengths (orange).

Displacement values were further increased when all force profiles and muscle lengths were included in the computation (mean ± SD: 8.6 ± 2.4 pps), compared with that of within force profile (*P* < 0.0001; Tukey‐adjusted) and across force profiles (*P* < 0.0001; Tukey‐adjusted). Note that motor unit data were not categorized or averaged prior to performing any statistical analysis; however, for clarity, we report the median displacement values for each subject in Figure 6b. The range and cumulative distribution of displacement values across the three condition categories are shown in Figure 6c,d.

**FIGURE 7:**
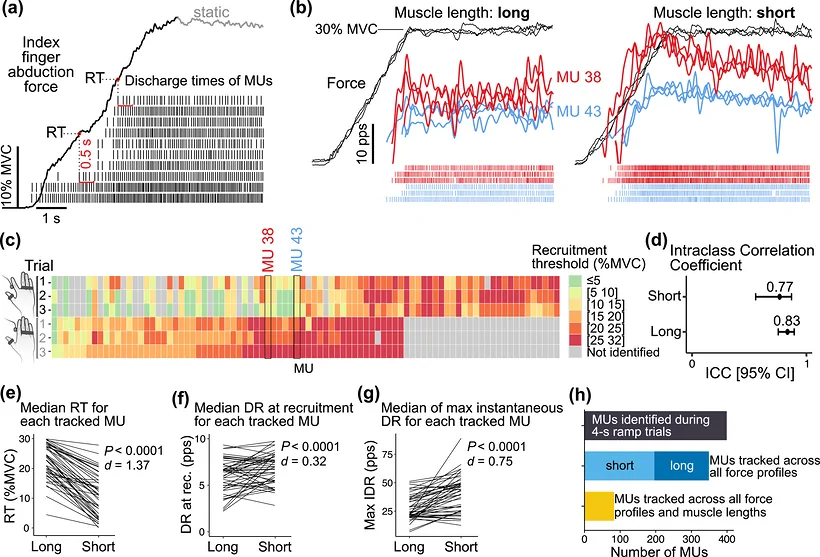
Recruitment thresholds and discharge rates of motor units during 4 s ramp contractions at long and short muscle lengths. (a) Recruitment threshold was defined as the force (%MVC) at which the first action potential was discharged within a 0.5 s window where the coefficient of variation for interspike intervals was less than 50%. (b) Each panel displays the force (top), smoothed discharge rates (centre) and spike rasters (bottom) for the same pair of motor units recorded at long (left) and short (right) muscle lengths. (c) Recruitment thresholds for all tracked motor units at short (first three rows) and long (last three rows) muscle lengths. Each column shows data for a single motor unit. (d) Intraclass correlation coefficients (with 95% confidence intervals) indicate absolute agreement in motor unit recruitment thresholds during 4 s ramp trials at long and short muscle lengths. (e) Median recruitment threshold for each motor unit as a function of muscle length. (f) Median discharge rate at recruitment for each motor unit as a function of muscle length. (g) Median of maximum instantaneous discharge rate for each motor unit as a function of muscle length. *P*‐values show the mean effect of muscle length on the outcome variables. Cohen's *d*‐values show the magnitude of difference. (h) Number of identified motor units during 4 s ramp trials (top), tracked across 3 trials at long or short muscle length (centre), tracked across all force profiles and muscle lengths (bottom).

Example state‐space trajectories for pairs of motor units in Figure 5d show that changes in muscle length modulate common inputs to motor units. For clarity, a single force profile was displayed in each panel. In the bottom right panel, for example, the discharge rate trajectories for motor units 4 and 6 during a chirp are shown at short (blue) and long (red) muscle lengths. At the short muscle length, motor unit 4 reached its highest discharge rate during the slowest segment of the task compared with the faster segments. First, this observation is in contrast with most of our data and not present at long muscle lengths. Similar observations were recorded in several trials (Figure 7, bottom panel). Second, at the long muscle length, motor unit 4 was silent during the slowest segment of the task. This is a clear deviation from a monotonic input signal. In some cases, monotonicity was not meaningfully violated, and the displacement was relatively small. For example, in the top right panel of Figure 5d, while the motor unit responses differed between the muscle lengths during a 4 s ramp up, their activity was largely monotonic.

### 3.4 Recruitment and discharge rates of motor units across muscle lengths

Motor units followed orderly recruitment during 4 s ramp up contractions at both long and short muscle lengths (Figure 7). The level of absolute agreement for recruitment thresholds was moderate to good at short muscle length (ICC = 0.77 [95% CI: 0.56, 0.87]) and good at long muscle length (ICC = 0.83 [95% CI: 0.75, 0.89]). Tracked single motor units were consistently recruited at lower thresholds at short compared with the long muscle length (*P* < 0.0001, *d* = 1.37), with some exceptions observed in individual trials (Figure 7c). Muscle length also modulated motor unit discharge rates during 4 s ramp up: both the discharge rate at recruitment (*P* < 0.0001, *d* = 0.32) and the maximum instantaneous discharge rate (*P* < 0.0001, *d* = 0.75) were significantly greater at short compared with the long muscle length. However, the observed systematic change in motor unit recruitment and discharge rates between the two muscle lengths could also be partly attributed to the fact that submaximal forces were normalized using an MVC value obtained only at the long muscle length. MVC has been reported to decline by approximately 63% when the index finger is maximally abducted compared with the neutral position (Zijdewind & Kernell, 1994).

**FIGURE 8:**
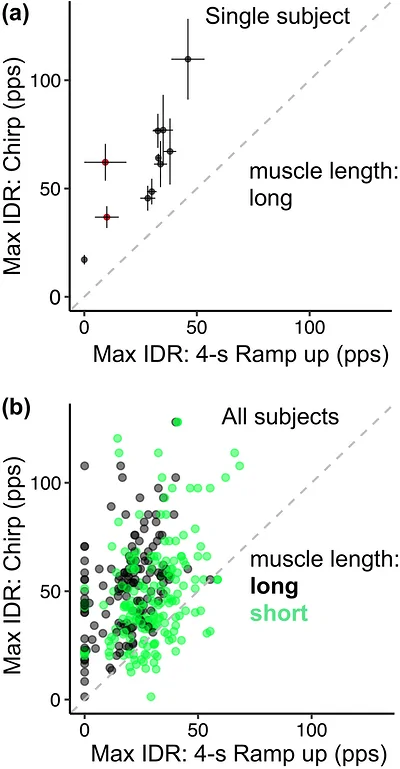
Maximum instantaneous discharge rates (IDR) of motor units during 4 s ramp up and chirp contractions. (a) Each data point shows the mean and standard error of maximum IDR for a single motor unit from one subject. Two motor units are coloured (red) to highlight that their maximum IDRs were similar during the 4 s ramp up but different during the chirp. (b) Each data point shows the maximum IDR for a single motor unit during the 4 s ramp up and chirp in a task block. Motor units reached higher maximum IDRs during the chirp at long muscle length, but some exceptions were observed at short muscle length. Many motor units were silent during the 4 s ramp but reached high rates during the chirp.

Next, we compared the maximum instantaneous discharge rates of motor units during 4 s ramp up and chirp contractions. Figure 8a shows the mean and standard error of maximum instantaneous discharge rate for individual motor units at long muscle length for one subject. Although some motor units had similar maximum instantaneous discharge rates during the 4 s ramp‐up, their discharge rates diverged during chirp. Motor units reached markedly higher discharge rates during the faster force profile (i.e., chirp). This observation was expected (Büdingen & Freund, 1976), but as shown in Figure 8b, some exceptions were observed only at short muscle length (green).

### 3.5 Motor unit modes

**FIGURE 9:**
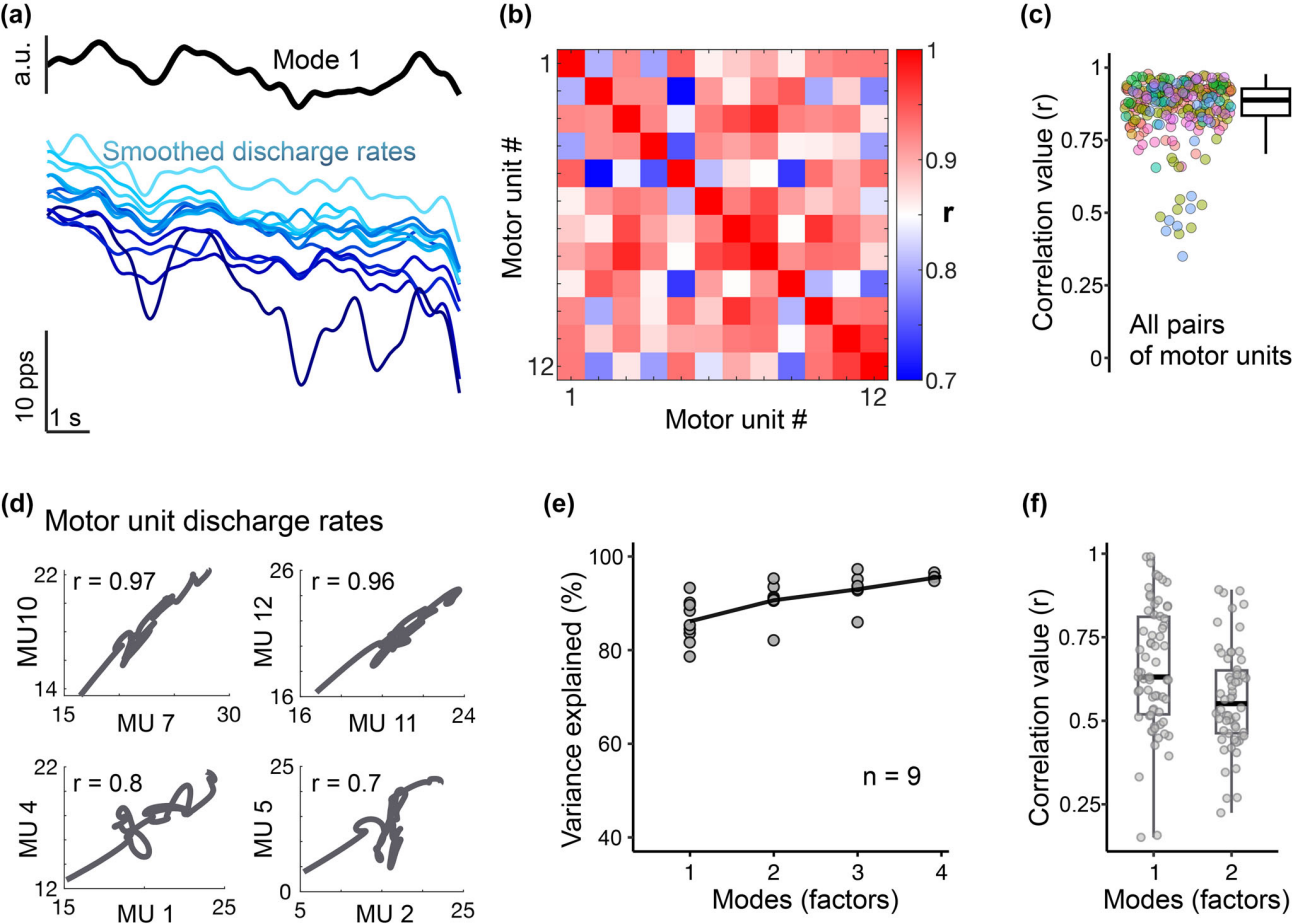
Motor unit modes. (a) Smoothed discharge rates of 12 motor units recorded during a 10 s static contraction at 30% MVC from a single representative trial. Each motor unit is shown with a different shade of blue. Factor analysis identified motor unit mode 1, which accounted for 88.4% of the shared variance (top black trace). Panels (b) and (d) use the same data. (b) Pairwise correlation matrix. (c) Correlation between the smoothed discharge rates of individual motor units, as an estimate of the proportion of common synaptic input. Each data point represents a pair of motor units colour‐coded by subject. (d) Rate–time plots for four representative pairs of motor units. (e) Cumulative sum of the common variance in motor unit discharge rates explained by each mode. The black line shows the average across subjects. On average, mode 1 explained 86.1% of the shared variance. (f) Correlations between the smoothed discharge rates of individual motor units and the first two motor unit modes for each subject. The box plots in panels (c) and (f) show the median value and the first to third quartiles.

We performed factor analysis on the smoothed discharge rates of motor units recorded during a 10 s static contraction, after determining the trial with the greatest number of identified motor units for each subject. The criteria for the factor analysis reduced the dataset to 9 of 10 subjects. Factor analysis identified 3.6 ± 2.2 modes from an average of 7.3 ± 3 motor units across subjects. This suggests that motor unit activity was driven by a limited number of shared inputs. As unique variances in discharge rates are not explained by modes, total variance explained can plateau before the number of modes reaches the number of motor units. We found that on average, motor unit mode 1 accounted for 86.1% of the shared variance (Figure 9f). Smoothed discharge rates of individual motor units were, on average, most strongly correlated with the motor unit mode 1 (Figure 9g). Discharge rates of some motor units correlated more strongly with mode 2 than with mode 1. As an estimate of the proportion of common synaptic input, we analysed the correlation between smoothed discharge rates of motor units (*n* = 245 pairs; Figure 9e). Discharge rates of motor units were highly correlated (*r* = 0.86 ± 0.11 [95% CI: 0.85, 0.87]).

**FIGURE 10:**
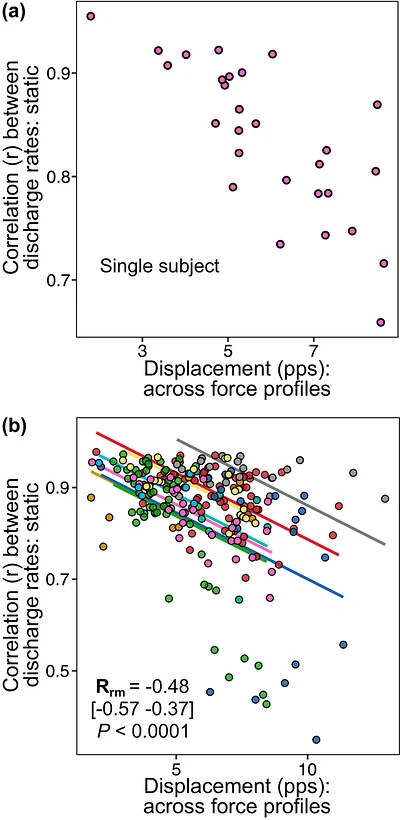
Association between motor unit displacement (across all force profiles) and correlation values of smoothed discharge rates (during 10 s static contractions). Each data point is a motor unit pair, colour‐coded by subject. (a) Data from an example subject. (b) Repeated‐measures correlation (*R*_rm_) with estimated common within‐subject slope; coloured lines show subject‐specific intercepts. *R*_rm_ coefficient, its 95% confidence interval and *P*‐value are reported in the panel.

Displacement (across all force profiles) was negatively associated with the pairwise correlation of motor unit discharge rates during 10 s static contractions. Figure 10 shows the *R*_rm_ coefficient, its 95% confidence interval and *P*‐value. These results provide support for the recently developed displacement metric (Marshall et al., 2022), suggesting its potential relevance in characterizing variations in common input structure.

## 4 DISCUSSION

**FIGURE 11:**
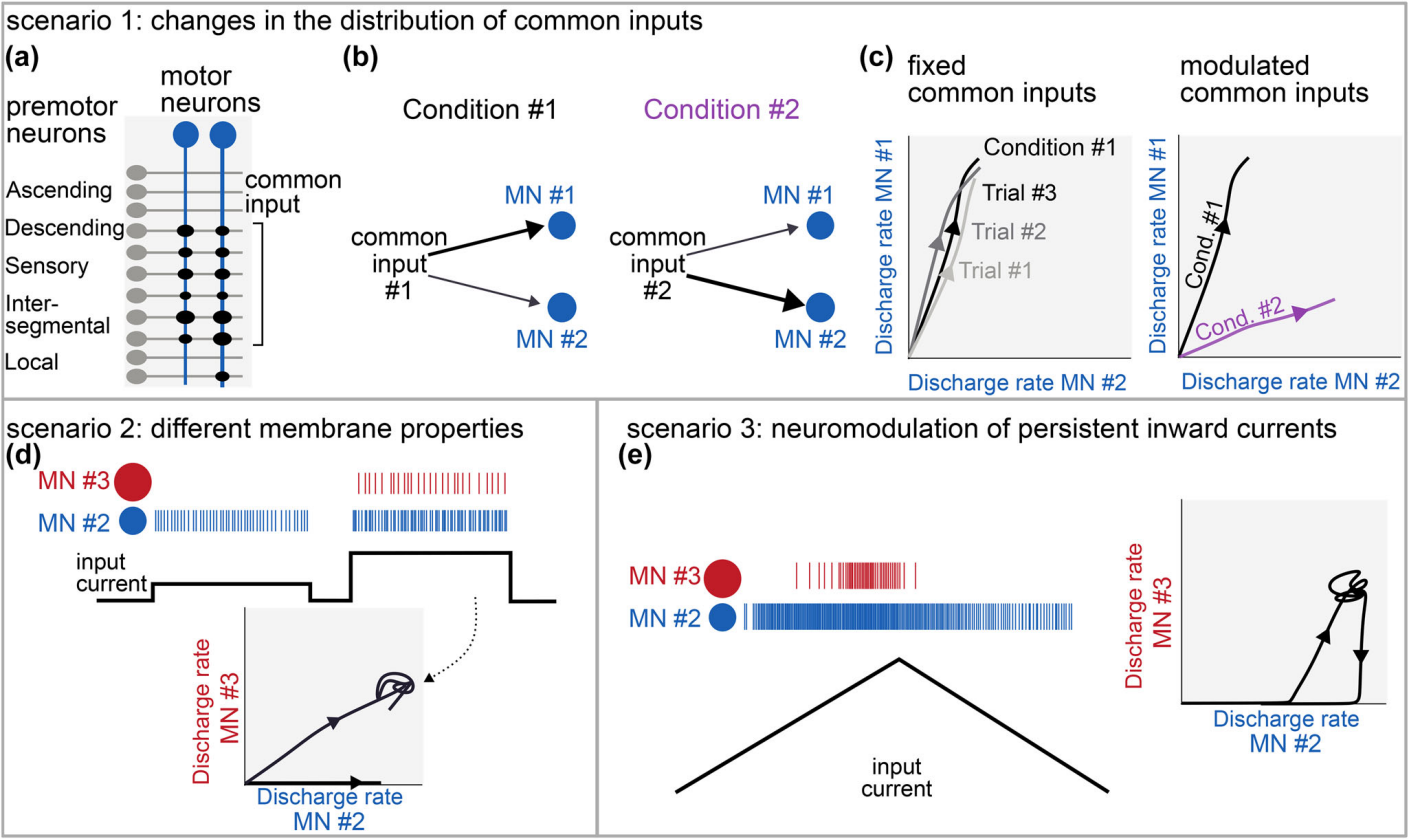
Summary. (a) Illustration of connectivity matrix between premotor and motor neurons. Common input refers to synaptic currents shared across multiple motor neurons. (b) Schematic illustration of distinct input patterns associated with two hypothetical conditions (e.g., different force profile or muscle length). (c) Left panel, state space activity of a motor unit pair. The activity is constrained to a space across the three trials within the same condition. Right panel, state space activity of the same motor unit pair under two different conditions. We defined each force profile (such as a 4 s ramp up) and muscle length (long and short) as a ‘condition’. The magnitude of displacement in state space trajectories reflects modulation of common inputs to motor units. (d) Membrane electrical properties of individual motor neurons dictate how they respond to synaptic inputs. Simply changing the amplitude of a common input current to a pair of motor neurons is enough to create displacement. (e) Neuromodulation of persistent inward currents can amplify and prolong synaptic inputs to motor neurons, causing violations in the monotonicity of discharge rate–time trajectories even under strictly common input.

In this study, we characterized how the distribution of common synaptic inputs to motor units is modulated by task demands and muscle length. We applied factor analysis to discharge rates during static contractions to estimate the proportion of common synaptic inputs. Factor analysis and pairwise correlation of discharge rates indicated a low‐dimensional control structure, with a single motor unit mode explaining most of the variance. We then computed displacement for motor unit rate–time trajectories within and across a range of conditions to estimate violations of monotonicity expected from fixed common inputs. Notably, our findings suggest that the common inputs to motor units are flexibly modulated by force profile and muscle length, yet is nearly fixed within each condition. Correlation and factor analysis provide metrics of covariance, whereas displacement captures violations of monotonic relationships between motor units. These complementary metrics (Figure 10) provide distinct insights into the neural control of motor units. Finally, we assessed recruitment thresholds and discharge rates of motor units as a function of muscle length across 4 s ramp up trials. Motor units were recruited in a consistent order, in accordance with the size principle, simplifying dimensionality of the motor system. Muscle length modulated both discharge rates and recruitment thresholds of motor units. A schematic summary of our main findings is provided in Figure 11.

Motor unit behaviour was remarkably consistent within a single task–muscle length condition. For the 4 s ramp up, 94.2% of all displacement values were less than 2 pps, suggesting that the motor system relies on reusing a common input structure when performing the same task. From the standpoint of the principle of motor abundance (Latash, 2012, 2020), which considers redundancy as vital for motor behaviour, much of the trial‐to‐trial noise present in the central nervous system is largely filtered out by the time it gets to the motor units. This further suggests that private inputs to individual motor units (Marshall et al., 2022), which would introduce much greater variability in motor output, is unlikely during voluntary muscle contractions. There is strong evidence that although groups of motor units within a pool may receive uncorrelated inputs, such inputs are shared, rather than private, with motor units from other pools (Farina & Negro, 2015; Hug et al., 2023; Lesser et al., 2024).

### 4.1 Task and muscle length related modulation of motor unit behaviour cannot be explained by a fixed common input

The original size principle, proposed by Henneman et al. (1965a, 1965b), suggests that all motor neurons within a pool receive the same synaptic input, regardless of premotor neuronal source (Clamann & Henneman, 1976; Henneman et al., 1965a; Somjen et al., 1965). An alternative view is that premotor neurons flexibly provide input to different subsets of motor neurons and allow selective recruitment (Formento et al., 2021; Marshall et al., 2022). Our findings suggest that the sensorimotor system controlling human finger abduction operates between these two views. Specifically, synaptic inputs to motor units appear constrained within each force profile, yet they are modulated by changes in force profile or muscle length, indicating shifts in the composition of presynaptic sources.

This observation is understandable. Our experiments involved a single action, finger abduction, performed across a range of force profiles varying in rate of force development: slow (4 s ramp), steady (static), rapid (0.5 s ramp) and a time‐varying profile characterized by contraction history (0–1.25 Hz chirp). It is plausible that the composition of premotor neuronal inputs to motor units differed across these conditions (Albert et al., 2020; Kornhuber, 1971). Rapid voluntary movements are initiated supraspinally, with few synaptic connections and no sensory feedback modification (Del Vecchio, 2023), whereas slow movements are tuned by various afferent inputs (Henry et al., 2025). Pyramidal tract neurons in the motor cortex discharge during both slow and fast voluntary movements (DeLong & Strick, 1974), but significantly reduce their discharge rate during static contractions (Baker et al., 2001; Slobounov et al., 2002). Most neurons recorded in the putamen discharge at high rates during the generation of slow ramp movements but only weakly during ballistic contractions (DeLong & Strick, 1974). Multiple premotor neuronal sources coding for force may suggest redundancy, but studies show differences in their force coding strategies (Glover & Baker, 2022; Kornhuber, 1971; Takei et al., 2026). Ultimately, common synaptic inputs, majorly routed by local interneurons, depolarize motor units in a size‐based order (Desmedt & Godaux, 1977; Lesser et al., 2024). Our data support this, but the noteworthy observation was that the rate–time trajectories for pairs of motor units significantly differed across force profiles, indicating that common input is not a fixed function; rather it is modulated by weighting of inputs from premotor neurons.

The role of local interneurons in shaping this modulation must be noted. In a recent study on non‐human primates, Takei et al. (2026) compared the contribution of corticomotoneurons and spinal local interneurons to the generation of hand muscle activity during a precision grip task. They found that the local interneuron activity had high temporal correlation with the target muscle activity, whereas corticomotoneuron activity had poor temporal correlation. Importantly, they reported that the lower dimensional control of motor units was mediated by the local interneurons, while cortical neurons were associated with higher dimensional control. Similar observations have also been reported in insect models (Lesser et al., 2024).

Alternatively, the observed displacement values could be partially explained by intrinsic properties of motor neurons. The membrane electrical properties of individual motor neurons, such as input resistance and membrane time constant, dictate how they respond to synaptic inputs. In a scenario where a low amplitude common input is sent to a pair of a small and a large motor unit, only the small motor unit will discharge action potentials. If the amplitude of the common input increases, however, the large motor unit will also start discharging. This would appear as a deviation from a monotonic manifold in state space, leading to displacement in discharge rate–time trajectories (Figure 11d). Second, descending brainstem serotonin and noradrenaline systems contribute to the neuromodulation of persistent inward currents, which plays a significant role in increasing cell excitability by amplifying and prolonging synaptic inputs to motor neurons (Heckman & Enoka, 2012). Therefore, even if the input to motor units is strictly common, monotonicity in discharge rate–time trajectories may still be violated (Figure 11e).

We aimed to alter sensory inputs by changing the joint angle (Tuthill & Azim, 2018). Accordingly, rate–time trajectories for motor unit pairs differed significantly between muscle lengths, suggesting that sensory feedback modulated the distribution of common synaptic inputs to motor units. Consistent with this, in a study of motor unit coherence, Cabral et al. (2024) found that changing the length of the tibialis anterior muscle from short to long resulted in a reduction in the common inputs to motor units in the 5–15 Hz bandwidth. They reported a reduction in the oscillations of dorsiflexion force within the same bandwidth and attributed these findings to altered sensory feedback and enhanced low‐pass filtering properties of the muscle at longer lengths. Moreover, existing reports of greater estimates of persistent inward currents and reflex amplitudes and lower recruitment thresholds at shorter muscle lengths might suggest a pattern of reduced inhibition (Dutt‐Mazumder et al., 2020; Frigon et al., 2007; Goreau et al., 2025; Nishimura & Nakajima, 2002). Similarly, in our data, tracked motor units were recruited at lower thresholds and reached higher discharge rates during 4 s ramp contractions at shorter muscle length. Both spinal and supraspinal neurons receive sensory feedback to achieve fine motor control (Gesslbauer et al., 2017); however, the association between the activity of sensory afferents and the dimensionality of motor control remains to be established.

### 4.2 Correlated discharge rates of motor units

Common synaptic inputs to groups of motor neurons result in temporal correlation between their action potential discharge times (Farina et al., 2016). The relative proportion of common inputs can be quantified by the strength of correlation between the discharge rates of motor units and modelling them into a number of factors, referred to as motor unit modes. Based on dimensionality reduction techniques, extensive research has shown that a small number of modes typically account for most of the variance in motor unit discharge rates (Cabral et al., 2025; Levine et al., 2023). For example, Weinman et al. (2024) found that the correlated discharge rates of the triceps surae muscles can be grouped into three motor unit modes during isometric contractions. Importantly, they reported that the composition of the motor unit modes was reorganized following a brief muscle stretch intervention, likely mediated by changes in afferent feedback, but not after a control task. Similarly, Del Vecchio et al. (2023) reported that two motor unit modes explained most of the common variance in motor unit discharge rates for two pairs of muscles (vastus lateralis and medialis; thenar and first dorsal interosseous). The analysis of their tracked motor units showed that around half of the motor units of the two knee extensor muscles were assigned to different modes across trials, but the proportion was smaller for the first dorsal interosseous (23%) and thenar (4%) muscles.

It is worth noting that the study by Del Vecchio et al. (2023) involved concurrent abduction of the index finger and flexion of the thumb. They showed that the motor units from the two hand muscles can be fully represented by a single mode, and only a few motor units were associated with both muscle‐specific neural modes. Similarly, based on our findings and previous reports, a single motor unit mode accounted for most of the covariation in the discharge rates of motor units from the same muscle (Cabral et al., 2025; Levine et al., 2023). This was further supported by the high correlation values between motor unit pairs from the same participant (95% CI: 0.85, 0.87), reflecting the strength of common synaptic inputs (Farina et al., 2016). Moreover, in a study involving multi‐joint isometric contractions, Hug et al. (2023) reported that even the motor units from distant muscles receive common inputs, such as biceps femoris and medial gastrocnemius, based on their correlation analysis of discharge rates.

In the muscle synergy literature, it is well established that extracting synergies from a single task cannot distinguish neural modularity from task‐based covariance (Kutch & Valero‐Cuevas, 2012; Russo et al., 2014; Tresch & Jarc, 2009). Muscle activations may covary within a single condition simply because the task demands it, and demonstrating that synergy structure is preserved across a sufficiently diverse set of tasks is considered a minimum requirement for inferring neural origin. This logic is largely absent in the factor analysis of motor unit discharge rates, where a single mode explaining most variance during a single task, static isometric contraction, is routinely interpreted as evidence for common input to motor units. It is possible that a sustained contraction at a fixed force level is precisely the condition where task constraints alone force motor unit discharge rates to covary. The present study's displacement analysis results make this point implicitly: if the input structure reorganizes across conditions, then the mode structure identified during static holds may not generalize. Performing factor analysis only within the least demanding condition and proposing that it indicates a fixed common synaptic input is the motor unit equivalent of extracting muscle synergies from a single reaching direction and calling them neural modules.

Our study has only explored a part of the neural control of voluntary movement landscape, and our results are not intended to generalize across limbs and actions. Displacement analysis could be biased for task blocks with very few motor units. Nevertheless, a large number of motor unit pairs (*n* = 2752 within force profile alone) were included in our analysis. Moreover, our study had a within‐subject, within‐motor unit repeated measures design. Another limitation of our study was that MVC was recorded only at the long muscle length to set relative target forces. Therefore, despite the large effect size (Figure 7e), our finding of a muscle length‐dependent systematic change in motor unit recruitment thresholds should be interpreted carefully. However, this limitation is unlikely to meaningfully affect the displacement analysis (Marshall et al., 2022). To demonstrate this, we provided examples in which monotonicity was not meaningfully violated despite muscle length‐dependent changes in motor unit responses: in Figure 5c and the top right panel of Figure 5d, while the motor unit recruitment thresholds and discharge rates differed between the muscle lengths, the displacement was relatively small. Finally, the remarkable within condition trial‐to‐trial stability of discharge rate–time trajectories could be due to that the index finger abduction is largely attributable to the first dorsal interosseus (Zijdewind & Kernell, 1994). In tasks involving multiple muscles, such as ankle plantar flexion, within condition variability in discharge rate–time trajectories for pairs of motor units may be greater.

In summary, our study supports the idea that integration of premotor neuronal inputs occurs to provide common synaptic inputs to groups of motor neurons. Common inputs are modulated to perform isometric muscle contractions across muscle lengths and force profiles varying in rate of force development. The common input structure is maintained to perform a single force profile, as our observations showed that the rate–time trajectories of motor unit pairs followed consistent, largely monotonic patterns with no substantial violations across trials, within each force profile. These findings highlight that common input, a key feature in the neural control of motor units, is subject to substantial modulation. We propose that future studies should focus more on (1) the ability to modulate common inputs to motor units in neurological conditions, such as stroke and amyotrophic lateral sclerosis, and (2) the functions of premotorneuronal sources coding for force. Finally, this work highlights the importance of analysing motor unit data using various complementary metrics to gain insights into the neural control of motor units.

## AUTHOR CONTRIBUTIONS

All experiments were performed at NEU, Movement Neuroscience Laboratory. Abdulkerim Darendeli designed the experiments, collected and analysed the data, and prepared the figures. All authors interpreted the data and contributed to drafting or critically revising the manuscript. All authors approved the final version of the manuscript, agreed to be accountable for all aspects of the work in ensuring that questions related to the accuracy or integrity of any part of the work are appropriately investigated and resolved, and all persons designated as authors qualified for authorship, and all those who qualify for authorship are listed.

## CONFLICT OF INTEREST

The authors declare no conflict of interest, financial or otherwise.

## GENERATIVE AI STATEMENT

No AI tools were used in the preparation of this manuscript.

## Data Availability

Data for this study are publicly available at https://doi.org/10.6084/m9.figshare.32509461.
